# Four‐fold increased mortality rate in patients with Wilson's disease: A population‐based cohort study of 151 patients

**DOI:** 10.1002/ueg2.12452

**Published:** 2023-08-25

**Authors:** Fredrik Åberg, Ying Shang, Rickard Strandberg, Axel Wester, Linnea Widman, Hannes Hagström

**Affiliations:** ^1^ Transplantation and Liver Surgery Helsinki University Hospital and University of Helsinki Helsinki Finland; ^2^ Department of Medicine Karolinska Institutet Stockholm Sweden; ^3^ Division of Hepatology Department of Upper GI Karolinska University Hospital Stockholm Sweden

**Keywords:** cardiovascular, death, liver transplantation, mortality, neurologic, neuropsychiatric, OLT, psychiatric, Wilson

## Abstract

**Background and Aims:**

Few studies have investigated mortality rates in patients with Wilson's disease and compared these to the general population. Here, we examined several clinical outcomes (including cardiovascular, psychiatric, neurologic conditions) in a population‐based study of patients with Wilson's disease.

**Method:**

We used nationwide registers to identify all patients with a first diagnosis of Wilson's disease between 2002 and 2020 in Sweden. Each patient was matched by age, sex, and municipality with up to 10 reference individuals from the general population. Validated registers were used to investigate outcomes up to 19 years after baseline in patients and reference individuals.

**Results:**

We identified 151 patients with Wilson's disease matched with 1441 reference individuals. Median age at baseline was 26 years (IQR 17–42) and 50% were males. During a median follow‐up of 6.6 years (IQR 2.9–12.9), 10 (6.6%) patients with Wilson's disease died compared with 31 (2.2%) reference individuals. This translated to a hazard ratio (HR) of 3.8 (95%CI = 1.8–8.1). Mortality was higher among Wilson's disease patients with baseline neuropsychiatric diagnoses (HR 7.9, 95%CI = 2.9–21.8). Cumulative mortality over 10 years was 9.3% (95%CI = 5.0–16.8) in Wilson's disease, compared to 2.4% (95%CI = 1.6–3.6) in reference individuals. We observed significantly elevated risks in the Wilson's disease group for incident cardiovascular disease, and incident psychiatric and neurological conditions when considering liver transplantation or death from other causes as competing events.

**Conclusion:**

In this large population‐based cohort study, patients with Wilson's disease had an almost four‐fold increased mortality rate compared with matched individuals from the general population.

## Graphical abstract

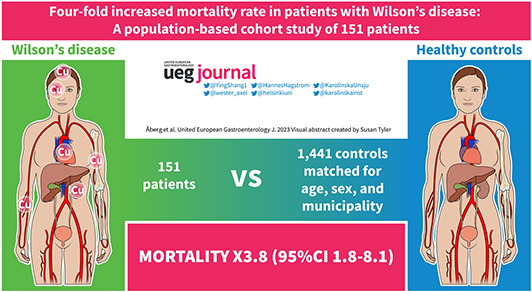

**Key summary**

**Summarise the established knowledge on this subject**

- Wilson's disease is a rare condition and data on long‐term outcomes come mainly from specialized clinics
- Increased risks for outcomes beyond those related to liver or neuropsychiatric diseases have previously been suspected

**What are the significant and/or new findings of this study?**

- In a population‐based study of 151 Wilson's disease patients, overall mortality was 3.8‐fold compared to matched reference individuals
- Mortality was particularly high among the Wilson's disease patients with baseline neuropsychiatric conditions
- Increased risk was observed for incident cardiovascular disease
- During the year 2020, the most common Wilson's disease medication was trientine (83%)

## INTRODUCTION

Wilson's disease is a rare genetic disorder of copper metabolism caused by mutations in the gene encoding the copper transporter ATP7B.^1^ Defective biliary excretion of copper leads to copper accumulation in the body. In most populations, the estimated prevalence of clinical Wilson's disease is around 1/30,000.^2^ The disease usually manifests between childhood and early adulthood. The clinical presentation is highly variable, but predominantly involves acute or chronic liver disease and neuropsychiatric disorders. Medical treatment for Wilson's disease currently consists of chelating agents (d‐penicillamine or trientine) and zinc, but there is a large variation in treatment regimens used in real‐world practice.

A limited number of studies report long‐term outcomes in Wilson's disease, and these studies are largely based on data from specialized clinics,^3^, ^4^, ^5^, ^6^, ^7^, ^8^, ^9^ which leaves some potential for referral bias. Many of these studies include cases diagnosed in the 1960s and 1970s,^3^, ^4^, ^7^, ^9^ when treatment regimens were less established and may not reflect the prognosis in patients diagnosed today. To our knowledge, there are no studies with nationwide coverage of all diagnosed cases of Wilson's disease in a country. In addition, copper accumulation affects several organs, but few studies have evaluated clinical outcomes beyond those related to the liver or central nervous system. Hence, modern data are lacking in outcomes in Wilson's disease, including mortality, the need for liver transplantation and the type of medication most commonly used.

Here, we used nationwide electronic healthcare registers to identify all cases in Sweden newly diagnosed with Wilson's disease in inpatient or outpatient care from 2002 and forward and compared several long‐term outcomes with matched general population reference individuals. We also investigated the most commonly used Wilson's disease medications during the year 2020.

## MATERIAL AND METHODS

We used the Swedish National Patient Register (NPR) to identify all individuals diagnosed with Wilson's disease in Sweden from 1 January 2002 until 31 December 2020. The NPR contains administrative coding from all persons discharged from any hospital in Sweden. Since 2001, the NPR has also included data on all outpatient visits in specialized care. The NPR's positive predictive value for most chronic diseases ranges between 85% and 95%^10^ and is >90% for diagnoses associated with cirrhosis, including hepatocellular carcinoma (HCC).^11^

**FIGURE 1:**
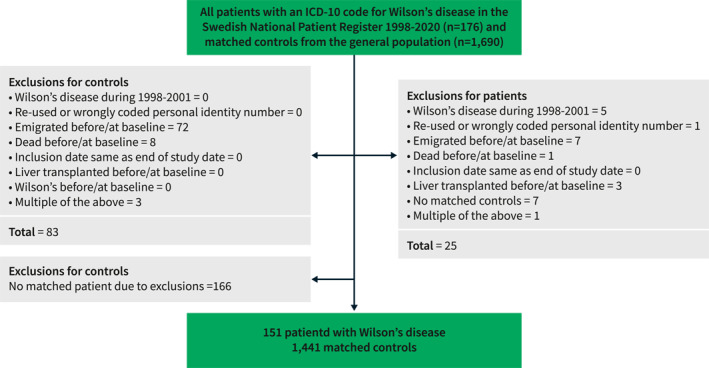
Flowchart of inclusion and exclusion criteria.

The presence of Wilson's disease was defined by the International Classification of Disease (ICD) code E83.0B (ICD‐10). The start of follow‐up (study baseline) was the first time‐point of ICD coding for Wilson's disease in the NPR. We excluded individuals meeting any of the following criteria at baseline: re‐used personal identity numbers, a diagnosis of Wilson's disease between 1998 and 2001 (this was done to exclude those with prevalent Wilson's disease at the start of the study period to ensure identification of only newly diagnosed patients), formally emigrated from Sweden, previously liver transplanted, or other administrative reasons (Figure 1).

Each patient with Wilson's disease was compared with up to 10 reference individuals from the general population and matched for age, sex and municipality at baseline. Reference individuals were identified through cross‐linking with the Total Population Register, which contains data on the date of birth, migration, death and other demographic parameters. This register is often used to link study participants to matched reference individuals for comparisons.^12^ Matching was done with direct matching using the central Swedish authority Statistics Sweden, based on age, sex and municipality at the date of first diagnosis of Wilson's disease. Data on previous liver transplantation and previous diagnoses of neurologic or psychiatric conditions were obtained from the NPR based on ICD‐codes (definitions in Supplementary Table 1).

The primary outcome was overall mortality, obtained from the National Causes of Death Register. We also analyzed a composite endpoint of death or liver transplantation. The National Causes of Death Register comprises data on all deaths in Sweden through a two‐step process. First, a death certificate in which a physician confirms the death is sent to the Swedish tax office. This certificate must be completed before a burial can be authorized. The second step entails a report of the cause of death filled in by a physician and sent to the National Board of Health and Welfare.^13^ Secondary outcomes included liver transplantation, liver‐related death, cardiovascular events, atrial fibrillation, HCC, cholangiocellular carcinoma, non‐hepatic cancers, end‐stage kidney disease, psychiatric and neurologic conditions, bone fractures, and parathyroideal disease. These secondary outcomes were selected based on previous research on suspected elevated risks in Wilson's disease.^14^ The outcomes were defined by ICD‐10 codes as shown in detail in Supplementary Table 1. The secondary outcomes were sourced from the Swedish inpatient and outpatient registers, cancer register, and Cause of Death register (liver‐related death as main cause of death). Follow‐up was until death, emigration, liver transplantation, occurrence of the investigated event, or 31 December 2020, whichever occurred first.

To validate the positive predictive value of the ICD‐10 code for Wilson's disease (E83.0B) in the national registers, we performed a manual chart review of all Wilson's disease cases diagnosed at the Karolinska University Hospital between 2010 and 2020 (*n* = 26).

To explore the types of Wilson's disease medications currently used, we sourced the Prescribed Drug Register^15^ among all Wilson's disease patients alive on 31 December 2020 for dispensations during the year 2020 of trientine (ATC‐code A16AX12), penicillamine (M01CC01), or zinc (A12CB). To ascertain the number of Wilson's disease patients who had received no Wilson's disease medications during the year 2020, we considered the following criteria: alive on 31 December 2020 without a liver transplant, living in Sweden, and having at least one registry record of the Wilson's disease diagnosis during the year 2020.

### Statistical methods

Continuous data are presented as median and 25^th^ and 75^th^ percentiles (IQR), and categorical variables as frequencies and percentages. The Wilcoxon rank‐sum test was used to investigate differences between groups for continuous variables and the Pearson's χ2 test for categorical variables. We calculated incidence rates per 1000 person‐years of follow‐up for the outcomes. Cumulative incidences for overall mortality were estimated using the Kaplan‐Meier method. Cox regression was used for comparing overall mortality within the matched strata of Wilson's disease patients and reference individuals. Within the Wilson's disease group, we constructed a Cox regression model with the year of diagnosis as independent variables to analyze temporal changes in overall mortality, adjusting for age and sex.

For the secondary outcomes, we used Fine and Gray competing‐risk regression (STATA: stcrreg) and the Aalen‐Johansen method (STATA: stcompet) to compare the rates and risks of outcomes in patients and reference individuals. In these analyses, liver transplantation and non‐outcome mortality were considered competing‐risk events (e.g. when investigating cardiovascular disease outcomes, non‐cardiovascular mortality was considered a competing event). Individuals with the outcome of interest at or before the baseline were further excluded from the respective analyses.

A 2‐tailed *p*‐value<0.05 was considered statistically significant. All analyses were performed using STATA version 16.1 and *R* version 4.1.2.

## RESULTS

We identified 151 patients with a record of a Wilson's disease diagnosis in Sweden between 2002 and 2020 (Figure 1). The median age was 26 years, 50% were men, 68% were born in Sweden, 17% had a neurologic diagnosis at or before the baseline, and 24% had a psychiatric diagnosis at or before baseline (Table 1). Baseline characteristics were similar between the patients born in Sweden and those born outside Sweden, except for a higher education level among those born outside Sweden (Table 1). The 151 patients with Wilson's disease were compared to 1441 matched general population reference individuals.

**TABLE 1:** Baseline characteristics of patients with Wilson's disease and matched general population reference individuals.

	Wilson's disease (*N* = 151)	Reference individuals (*N* = 1441)	*p*	Wilson's disease patients born in Sweden (*N* = 102)	Wilson's disease patients born outside Sweden (*N* = 49)	*p*
Follow‐up (years)	6.57 (2.88–12.90)	7.54 (3.83–13.64)	0.08	6.71 (3.30–12.77)	6.06 (1.75–13.79)	0.31
Men	76 (50.3%)	728 (50.5%)	0.96	48 (47.1%)	28 (57.1%)	0.25
Age at baseline, years	26 (17–42)	26 (17–41)	0.72	27 (16–44)	26 (19–34)	0.68
<10	14 (9.3%)	139 (9.6%)		13 (12.7%)	1 (2.0%)	
10–19	36 (23.8%)	356 (24.7%)		23 (22.5%)	13 (26.5%)	
20–29	35 (23.2%)	338 (23.5%)		17 (16.7%)	18 (36.7%)	
30–39	23 (15.2%)	212 (14.7%)		14 (13.7%)	9 (18.4%)	
40–49	19 (12.6%)	175 (12.1%)		16 (15.7%)	3 (6.1%)	
50 +	24 (15.9%)	221 (15.3%)		19 (18.6%)	5 (10.2%)	
Period of inclusion			1.00			0.71
2002–2005	27 (17.9%)	256 (17.8%)		20 (19.6%)	7 (14.3%)	
2006–2010	36 (23.8%)	337 (23.4%)		22 (21.6%)	14 (28.6%)	
2011–2015	35 (23.2%)	328 (22.8%)		23 (22.5%)	12 (24.5%)	
2016–2020	53 (35.1%)	520 (36.1%)		37 (36.3%)	16 (32.7%)	
Country of birth			<0.001			<0.001
Nordic countries	104 (68.9%)	1286 (89.2%)		102 (100%)	2 (4.1%)	
Europe (non‐Nordic)	12 (7.9%)	33 (2.3%)		0	12 (24.5%)	
Other	35 (23.2%)	122 (8.5%)		0	35 (71.4%)	
Education			0.52			
<9 years	24 (15.9%)	205 (14.2%)		18 (17.6%)	6 (12.2%)	0.01
9–12 years	57 (37.7%)	618 (42.9%)		44 (43.1%)	13 (26.5%)	
>12 years	49 (32.5%)	438 (30.4%)		24 (23.5%)	25 (51.0%)	
Missing	21 (13.9%)	180 (12.5%)		16 (15.7%)	5 (10.2%)	
Neurologic diagnosis before or at baseline	26 (17.2%)	94 (6.5%)	<0.001	17 (16.7%)	9 (18.4%)	0.80
Psychiatric diagnosis before or at baseline	36 (23.8%)	144 (10.0%)	<0.001	37 (36.3%)	17 (34.7%)	0.90

*Note*: Results are presented as *n* (%) or median (IQR).

During a median follow‐up of 6.6 years (IQR 2.9–12.9, range 0.2–18.8), there were 10 deaths (6.6%) in the Wilson's disease group. During a median follow‐up of 7.5 years (IQR 3.8–13.6, range 0.7–18.8) in the reference group, there were 31 deaths (2.2%). The median age at death was 64 years among both Wilson's disease patients and reference individuals. A comparison of baseline characteristics between patients with Wilson's disease who died or were alive at the end of follow‐up is presented in Supplementary Table 2. Causes of deaths are specified in Supplementary Table 3.

**FIGURE 2:**
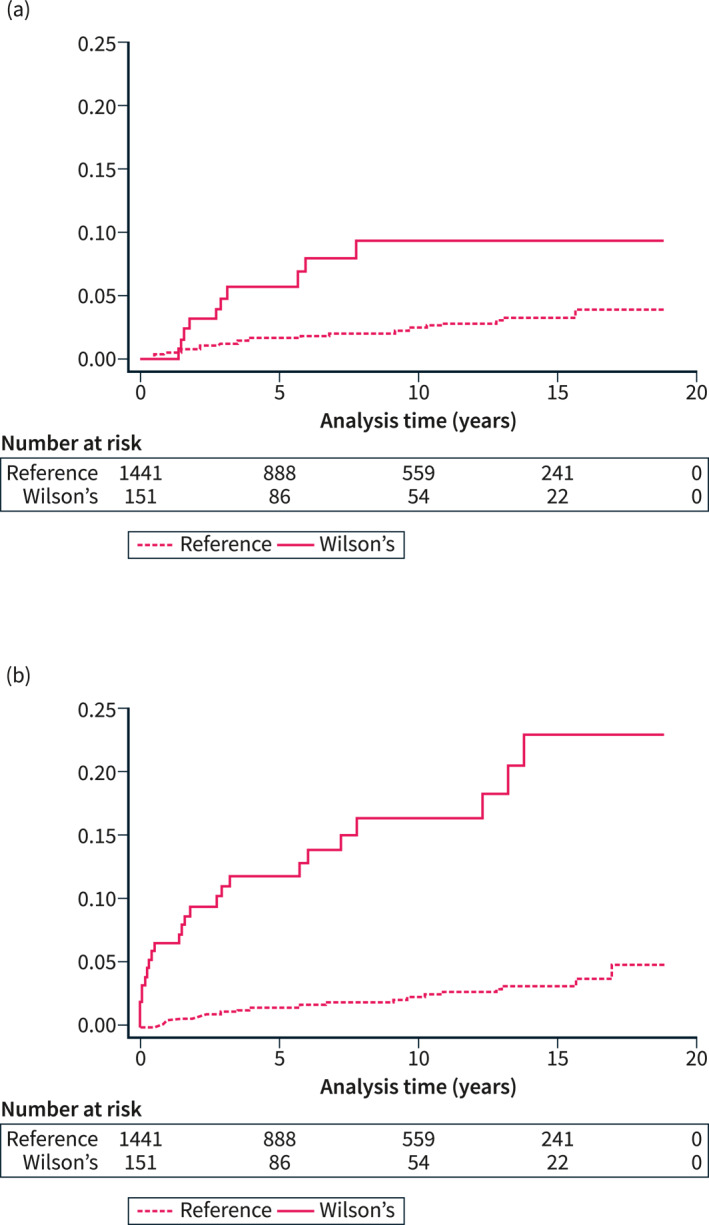
Cumulative all‐cause mortality (panel A) among Wilson's disease patients and reference individuals, and the cumulative incidence of a composite endpoint of either death or liver transplantation (panel B).

The overall mortality rate per 1000 person‐years was 8.53 among Wilson's disease patients and 2.53 among reference individuals, which translated into a hazard ratio (HR) of 3.84 (95% confidence interval (CI) 1.84–8.05) (Table 2). Kaplan‐Meier estimates for cumulative overall mortality in patients with Wilson's disease were 0% over 1 year, 5.6% over 5 years and 9.3% over 10 years (Table 3 and Figure 2). The year of diagnosis was not significantly associated with overall mortality (HR 0.97, 95% CI 0.82–1.13) by Cox regression analysis adjusted for age and sex.

**TABLE 2:** Overall mortality outcomes in Wilson's disease patients and reference individuals.

	Wilson's disease			Reference individuals			HR (95%CI)
	*N*	Deaths, N (%)	Mortality per 1000 person‐years (95% CI)	*N*	Deaths, *N* (%)	Mortality per 1000 person‐years (95% CI)	
All	151	10 (6.6)	8.53 (4.59–15.85)	1441	31 (2.2)	2.53 (1.78–3.60)	3.84 (1.84–8.05)
Men	76	5 (6.6)	8.04 (3.35–19.31)	728	13 (1.8)	1.99 (1.16–3.43)	3.53 (1.26–9.92)
Women	75	5 (6.7)	9.09 (3.78–21.84)	713	18 (2.5)	3.15 (1.98–5.00)	4.22 (1.46–12.14)
Age <20 years at baseline	50	0 (0.0)	0	495	2 (0.4)	0.58 (0.14–2.30)	‐
Age ≥20 years at baseline	101	10 (9.9)	11.83 (6.37–22.00)	946	29 (3.1)	3.31 (2.30–4.76)	4.17 (1.97–8.82)
Wilson's disease, born in Sweden	102	8 (9.4)	9.80 (4.90–19.60)	980	22 (2.2)	2.63 (1.73–3.99)	3.95 (1.73–9.02)
Wilson's disease, born outside Sweden	49	2 (4.1)	5.62 (1.41–22.47)	461	9 (2.0)	2.33 (1.21–4.48)	3.47 (0.67–18.00)
Neuropsychiatric condition	50	7 (14.0)	20.72 (9.88–43.47)	483	14 (2.9)	3.79 (2.25–6.40)	7.87 (2.85–21.75)
No neuropsychiatric condition	101	3 (3.0)	3.60 (1.16–11.15)	958	17 (1.8)	1.99 (1.24–3.20)	1.77 (0.52–6.07)

Abbreviations: CI, confidence interval; HR, hazard ratio.

**TABLE 3:** Cumulative overall mortality in Wilson's disease patients and reference individuals.

Cumulative mortality	Wilson's disease patients			Reference individuals		
	1 year	5 years	10 years	1 year	5 years	10 years
All	0.0 (−)	5.6 (2.7–11.5)	9.3 (5.0–16.8)	0.4 (0.2–0.9)	1.5 (1.0–2.3)	2.4 (1.6–3.6)
Men	0.0 (−)	6.2 (2.4–15.6)	8.6 (3.6–19.9)	0.3 (0.1–1.1)	1.5 (0.8–2.8)	1.8 (1.0–3.4)
Women	0.0 (−)	5.1 (1.7–15.0)	10.0 (4.2–22.7)	0.6 (0.2–1.5)	1.4 (0.7–2.7)	3.1 (1.8–5.2)
Age <20 years	0.0 (−)	0.0 (−)	0.0 (−)	0.0 (−)	0.2 (0.0–1.6)	0.2 (0.0–1.6)
Age ≥20 years	0.0 (−)	8.1 (3.9–16.2)	12.7 (7.0–22.5)	0.6 (0.3–1.4)	2.1 (1.3–3.3)	3.3 (2.2–5.0)
Wilson's disease, born in Sweden	0 (−)	5.6 (2.4–12.9)	10.8 (5.4–20.9)	0.3 (0.1–1.0)	1.5 (0.9–2.5)	2.5 (1.5–4.0)
Wilson's disease, born outside Sweden	0 (−)	5.8 (1.5–21.5)	5.8 (1.5–21.5)	0.7 (0.2–2.0)	1.5 (0.7–3.2)	2.3 (1.1–4.8)
Neuropsychiatric condition	0 (−)	12.5 (5.4–27.5)	20.7 (10.0–39.7)	0.6 (0.2–1.9)	1.7 (0.8–3.6)	3.5 (1.9–6.4)
No neuropsychiatric condition	0 (−)	2.4 (0.6–9.3)	4.1 (1.3–12.3)	0.3 (0.1–1.0)	1.4 (0.8–2.4)	2.0 (1.2–3.4)

*Note*: Results are in per cent.

There were no deaths among patients with Wilson's disease with a first record of the diagnosis at ages <20 years (Table 2). Mortality was similar between men and women, and between Wilson's disease patients born in or outside Sweden. In the subgroup of Wilson's disease patients with a neurologic or psychiatric diagnosis at or before baseline (*N* = 50), the HR for mortality compared to their matched reference individuals was 7.87 (95% CI 2.85–21.75), higher than the corresponding HR among Wilson's disease patients without neurologic or psychiatric diagnoses (HR 1.77, 95% CI 0.52–6.07). This difference was formally tested with Wald statistics and was significant (*p* = 0.045).

**FIGURE 3:**
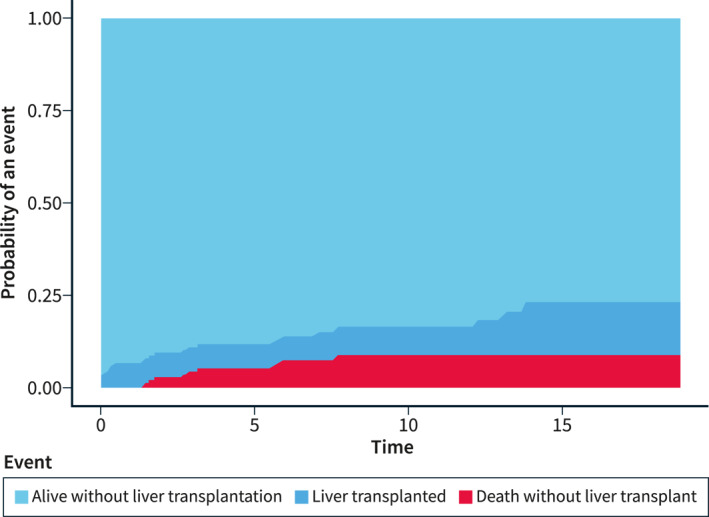
The cumulative probability of liver transplantation and death without liver transplantation as competing‐risk events in a multistate analysis among Wilson's disease patients.

Among patients with Wilson's disease, 14 (9.8%) underwent liver transplantation. Seven (50%) of these transplantations were performed within 3 months following the first record of a Wilson's disease diagnosis. As expected, by competing‐risk regression analysis, the incidence of liver transplantation was substantially higher among Wilson disease patients compared to the reference group (subdistribution HR [subdistribution hazard ratio (sHR)]137.2, 95% CI 17.9–1048.6), but there was only one case of liver transplantation among the reference individuals. For the composite endpoint of death or liver transplantation, the cumulative incidences in patients with Wilson's disease were 6.6% over 1 year, 11.9% over 5 years and 16.5% over 10 years (Figure 2). Figure 3 shows the cumulative probability of liver transplantation and death without a transplant in a multistate competing‐risk analysis.

For the composite endpoint of death or liver transplantation, patients with Wilson's disease had a HR of 8.93 (95% CI 5.10–15.65) compared to the reference individuals. In the subgroup of Wilson's disease with a neurologic or psychiatric diagnosis at or before baseline, the HR was 12.38 (95% CI 5.21–29.42). In the subgroup without such neurologic or psychiatric diagnoses, the HR was 6.99 (95% CI 3.31–14.79).

Regarding other secondary outcomes, we observed statistically significant elevated sHRs in the Wilson's disease group for incident cardiovascular disease (sHR 2.90, 95% CI 1.85–4.53), psychiatric diagnoses (sHR 2.11, 95% CI 1.36–3.29), and neurologic diagnoses (sHR 2.12, 95% CI 1.34–3.35), when considering liver transplantation or death from other causes as competing‐risk events (Table 4). The cumulative incidences of secondary outcomes are shown in Table 5.

**TABLE 4:** Incident secondary outcomes using Fine & Gray regression with liver transplantation and death from causes other than the event under study as competing‐risk events.

	Wilson's disease patients		Reference individuals		sHR (95%CI)
	Events/subjects	Incidence rate per 1000 person‐years	Events/subjects	Incidence rate per 1000 person‐years	
Liver‐related death	1/151	0.85 (0.12–6.06)	1/1441	0.08 (0.01–0.58)	9.54 (0.60–151.89)
Cardiovascular disease	26/122	32.23 (21.95–47.34)	92/1111	10.26 (8.36–12.59)	2.90 (1.85–4.53)
Atrial fibrillation	3/149	2.62 (0.85–8.13)	20/1412	1.68 (1.08–2.60)	1.38 (0.41–4.72)
Non‐hepatic cancer	5/145	4.46 (1.86–10.71)	54/1350	4.78 (3.66–6.24)	0.84 (0.33–2.11)
Hepatocellular carcinoma	1/150	0.86 (0.12–6.10)	1/1432	0.08 (0.01–0.58)	9.22 (0.55–154.79)
Cholangiocarcinoma	0/151	‐	0/1440	‐	‐
Chronic kidney disease	0/151	‐	0/1439	‐	‐
Psychiatric diagnoses	24/115	30.29 (20.30–45.19)	106/988	12.79 (10.57–15.47)	2.11 (1.36–3.29)
Neurologic diagnoses	23/125	25.12 (16.70–37.81)	102/1120	10.91 (8.99–13.25)	2.12 (1.34–3.35)
Bone fractures	22/134	23.10 (15.21–35.08)	132/1099	14.26 (12.02–16.91)	1.41 (0.91–2.20)
Parathyroideal disease	0/151	‐	0/1435	‐	‐

Abbreviations: CI, confidence interval; sHR, subdistribution hazard ratio.

**TABLE 5:** Cumulative incidence of secondary outcomes in Wilson's disease patients and reference individuals by competing‐risk analysis.

	Wilson's disease patients			Reference individuals		
	1 year	5 years	10 years	1 year	5 years	10 years
Liver‐related death	0.8 (0.1–3.9)	‐	‐	0.1 (0.0–0.5)	‐	‐
Liver transplantation	6.6 (3.4–11.3)	7.8 (4.1–13.2)	9.8 (4.9–16.6)	1.0 (0.1–4.8)	1.0 (0.1–4.8)	1.0 (0.1–4.8)
Cardiovascular disease	5.8 (2.5–10.9)	16.1 (9.7–23.8)	25.8 (16.8–35.8)	0.6 (0.3–1.3)	4.3 (3.1–5.8)	9.1 (7.1–11.4)
Atrial fibrillation	0.8 (0.1–3.8)	‐	‐	0.2 (0.1–0.6)	0.5 (0.2–1.1)	1.5 (0.8–2.6)
Non‐hepatic cancer	0.7 (0.1–3.5)	2.6 (0.7–6.9)	‐	0.7 (0.3–1.2)	2.1 (1.4–3.0)	4.8 (3.5–6.4)
Hepatocellular carcinoma	‐	‐	‐	‐	‐	‐
Cholangiocarcinoma	‐	‐	‐	‐	‐	‐
Chronic kidney disease	‐	‐	‐	‐	‐	‐
Psychiatric diagnoses	3.5 (1.1–8.1)	14.3 (8.2–22.2)	23.5 (14.8–33.3)	1.3 (0.7–2.2)	5.9 (4.5–7.6)	12.8 (10.3–15.5)
Neurologic diagnoses	4.8 (2.0–9.6)	11.5 (6.4–18.2)	20.2 (12.4–29.3)	1.1 (0.6–1.8)	5.4 (4.0–6.9)	10.5 (8.4–12.9)
Bone fractures	2.3 (0.6–5.9)	8.7 (4.4–14.7)	19.1 (11.7–27.9)	1.7 (1.1–2.6)	7.8 (6.2–9.7)	13.4 (11.1–15.9)
Parathyroideal disease	‐	‐	‐	‐	‐	‐

*Note*: Results are in per cent.

We identified 54 Wilson's disease patients who were alive on 31 December 2020 and that had dispensed Wilson's disease‐related medications during the year 2020. Among these 54 patients, 45 (83%) had dispensed trientine, 8 (15%) penicillamine, and 5 (9%) zinc during the year 2020. There were an additional 32 Wilson's disease patients who had received none of the above medications during the year 2020.

By manual chart review of all Wilson disease cases diagnosed at the Karolinska University Hospital between 2010 and 2020 (*n* = 26), the positive predictive value of the ICD‐10 code E83.0B was 100%.

## DISCUSSION

In this nationwide register‐based study, patients with Wilson's disease exhibited an almost 4‐fold increased risk of death compared with matched reference individuals from the general population. When considering a composite endpoint of either death or liver transplantation, the risk was 9‐fold in the patients with Wilson's disease compared with reference individuals. There was no improvement in mortality rates in the Wilson's disease group over the study period (2002–2020). Moreover, in the subgroup of patients with baseline neuropsychiatric conditions, the mortality risk was nearly eight‐fold compared with population reference individuals. We further observed increased rates for incident cardiovascular diseases and neuropsychiatric diseases among patients with Wilson's disease.

In our study, the cumulative mortality over 10 years after a diagnosis of Wilson's disease was 9.3%. A limited number of recent studies have reported long‐term cumulative mortality rates in Wilson's disease. Beinhardt and colleagues reported 10‐year mortality of 4.1% in their study of 229 Wilson's disease patients identified from Austrian tertiary referral centers during 1961–2013.^3^ Bruha and colleagues observed 10‐year mortality of 3% among 117 Wilson's disease patients identified from a general teaching hospital in Prague, Czech Republic, during 1965–2008.^4^ In contrast, Svetel and colleagues reported a 15‐year mortality as high as 23% among 142 Wilson's disease patients identified from several clinics in Belgrad, Serbia, during 1980–2007.^8^ Furthermore, previous studies reported that mortality among Wilson's disease patients was either similar^3^, ^4^, ^7^ or slightly elevated^5^ compared to the mortality in the general population, but matching of the comparator group was often inadequate or unclear in these studies.

We observed no deaths among Wilson's disease patients diagnosed at ages <20 years, likely because of insufficient length of follow‐up. In parallel, a higher proportion of pediatric patients in some of the previous studies^3^ might have contributed to the overall lower mortality rates observed in those studies compared with ours. The fact that one third of all patients in our study diagnosed with Wilson's disease was born outside Sweden reflects the rarity of the disease among Scandinavians.

Strengths of our study include the nationwide coverage of the registries used to identify cases. This mitigates and even eliminates selection bias. Our study thereby provides a comprehensive picture of long‐term outcomes in a large cohort of Wilson's disease patients in real‐world practice in entire Sweden. The inclusion of patients only from the modern era (2002–2020) and comparison to matched population controls are additional study strengths. The national, population‐based registers used for ascertaining exposure and outcome status are validated and are a source of high‐quality data.^10^, ^12^, ^13^ Furthermore, by manual chart review, a register record of Wilson's disease had a positive predictive value of 100%.

The main limitations include a lack of granular data on clinical characteristics such as the stage of disease at diagnosis, adequacy/quality of treatment, and patient compliance. With the lack of these data, it is impossible to conclude the reasons for the increased overall mortality observed in the Wilson's disease group. Nonetheless, the rate of liver transplantation of 9.8%, as a rough surrogate for medical treatment failure, was no higher than the rate reported in a large Austrian study (13.2%).^3^ In addition, 50% of all liver transplantations were performed within 3 months after the first record of Wilson's disease, which likely reflects acute liver failure or decompensated cirrhosis present at diagnosis. Moreover, Sweden has a high level of healthcare coverage and drug reimbursement, which makes expensive therapies such as trientine affordable to patients. Sweden also has a national knowledge center for Wilson's disease in Uppsala that provides consultations across the country. These aspects might point to a high standard of care of Wilson's disease in Sweden.

The small number of deaths (*n* = 10) led to wide confidence intervals, so estimates need to be interpreted with caution. The few outcome events and lack of granular treatment data also precluded robust pharmacoepidemiological association analyses. The rarity of outcomes calls for more international collaborations and meta‐analyses in the field.

Copper accumulation in Wilson's disease can affect multiple organs beyond the liver and the central nervous system. Previous studies have suggested a broad range of clinical manifestations, including cardiac, renal, osteoarticular and endocrinological involvement.^14^ Current treatment guidelines do not explicitly consider such manifestations as part of the evaluation of treatment efficacy.^16^, ^17^ There is also a paucity of evidence regarding the absolute risks and risks relative to the population of such manifestations. We found an increased risk of incident cardiovascular disease, and neuropsychiatric diagnoses, but no significantly elevated risks for the other preselected outcomes. Regarding cardiovascular disease risk, further study is needed to elaborate on whether this increased risk is due to the Wilson's disease itself, its treatment, or possible detection bias in that Wilson's disease patients under close surveillance might be more likely to be picked up for such diseases. However, a possible detection bias does not explain the increased overall mortality in the Wilson's disease group. Granular data on common cardiovascular risk factors such as cholesterol and smoking were unavailable. Further, risk estimates are based on relatively few outcomes with wide confidence intervals, so these should be interpreted with some caution.

Buksińska‐Lisik and colleagues previously performed extensive cardiovascular investigations on patients with Wilson's disease undergoing treatment and found only minor heart involvement such as mild left ventricular hypertrophy and subclinical changes in diastolic function.^18^ Nonetheless, that study included mainly young patients (mean age 33 years) who had received treatment already for an average of 4 years. In our study, there were more patients diagnosed at later ages, where the long‐lasting copper accumulation might presumably have caused more cardiovascular damage.

In the subgroup of Wilson's disease patients with a neurologic or psychiatric diagnosis at or before baseline (∼1/3 of all patients), the risk of death relative to population controls was amplified compared to the Wilson patients without such baseline neuropsychiatric diagnoses. Mortality estimates over 10‐year after the Wilson's disease diagnosis were 21% in those with baseline neuropsychiatric diagnoses and 4% in those without. At least one previous study has suggested that neurologic symptoms are associated with poorer compliance to therapy.^19^ Another plausible explanation could be that patients with neuropsychiatric diagnoses have more advanced disease and more extensive liver damage, leading to higher mortality. Buksińska‐Lisik and colleagues found that patients with neurologic Wilson's disease had more severe heart abnormalities than patients with hepatic Wilson's disease, speculating that this may be because neurologic Wilson's disease is often diagnosed at a later age with longer lasting whole‐body copper accumulation.^18^

In this modern series, most Wilson's disease patients (83%) received trientine during the year 2020. This is a substantially higher proportion than in previous studies, where a minority was reported to be on trientine therapy.^4^, ^5^, ^8^, ^20^ However, we did not perform a detailed analysis of treatment during the entire observation period and could not analyze the reasons for this frequent trientine use. Although trientine is considerably more expensive than penicillamine, both drugs are included in the national Swedish reimbursement system, which means that above an annual medication cost of around 230€, medications become fully subsidized to the patient.

In conclusion, in a population‐based cohort study, patients with Wilson's disease had an almost four‐fold increased rate of death compared with matched individuals from the general population. In addition, the risks for incident cardiovascular disease and incident psychiatric and neurological conditions were elevated.

## AUTHOR CONTRIBUTIONS

Study conception and design: Fredrik Åberg, Hannes Hagström. Acquisition of data: Hannes Hagström. Statistical analysis: Linnea Widman. Analysis and interpretation of data: All. Drafting of manuscript: Fredrik Åberg. Critical revision: All. Guarantor of article: Fredrik Åberg and Hannes Hagström. All authors approved the final version of the article, including the authorship list. Writing Assistance: None.

## CONFLICT OF INTEREST STATEMENT

The authors declare no conflicts of interest.

## ETHICS APPROVAL

The study was approved by the Regional Ethics Review Board in Stockholm (dnr 2017/1019‐31/1).

## PATIENTS CONSENT STATEMENT

Since this study included analyses of de‐identified data, written consent from participants was not required.

## PERMISSION TO REPRODUCE MATERIAL FROM OTHER SOURCES

Not applicable.

## CLINICAL TRIAL REGISTRATION

Not applicable.

## Supporting information

Supplementary Information S1

## Data Availability

Data are subject to personal information protection regulations and are not publicly available. Sharing of anonymized data will be considered on a case‐by‐case basis on request.
