# Supplementary material for: Four‐fold increased mortality rate in patients with Wilson's disease: A population‐based cohort study of 151 patients
Source: United European Gastroenterol J. 2023 Aug 25;11(9):852–60. doi: 10.1002/ueg2.12452 (PMC10637123; doi:10.1002/ueg2.12452)
Supplement: Supplementary file 1 — Supplementary Information S1 [file UEG2-11-852-s001.docx]

**Supplementary document**

**Supplementary table 1.** ICD-codes used for defining liver transplantation, neurologic and psychiatric conditions, as well as other secondary outcomes.

| **Outcome** | **ICD-10** |
| --- | --- |
| Liver transplantation | Z94.4 (or ICD-9: V42H)  Procedure codes: JJC00, JJC10, JJC20, DJ005, DJ006, JJC30, JJC40 (or ICD-9: 5200) |
| Liver-related death | K70-K77, R18, I850, I859, I982, I983, C220, E830B |
| Cardiovascular outcomes | I00-I99 |
| Atrial fibrillation | I48 |
| Non-hepatic cancers | C00-C97, excluding C22 |
| Hepatocellular carcinoma | C22.0 |
| Cholangiocellular carcinoma | C22.1 |
| Renal-replacement therapy or kidney transplantation | Z94.0, Z49.1, Z49.2  Procedure codes: KAS00, KAS10, KAS20, DR016, DR024 |
| Psychiatric diagnosis | F00-F99 |
| Neurologic diagnosis | G00-G99 |
| Fractures | S02, S12, S22, S32, S42, S52, S62, S72, S82, S92, T02, T08, T10, T12, M80, M485, M495 |
| Parathyroideal disease | E20-E21 |

**Supplementary table 2.** A comparison of baseline characteristics between patients with Wilson’s disease who had died or were alive at end of follow-up.

|  | **Died (N=10)** | **Alive (N=141)** | ***p*** |
| --- | --- | --- | --- |
| Follow-up (years) | 2.80 (1.57-5.68) | 6.85 (3.18-13.26) | 0.015 |
| Men | 5 (50.0%) | 71 (50.4%) | 0.98 |
| Age at baseline, years | 60 (46-72) | 25 (17-39) | <0.001 |
| <10 | 0 (0.0%) | 14 (9.9%) | <0.001 |
| 10-19 | 0 (0.0%) | 36 (25.5%) |  |
| 20-29 | 0 (0.0%) | 35 (24.8%) |  |
| 30-39 | 2 (20.0%) | 21 (14.9%) |  |
| 40-49 | 1 (10.0%) | 18 (12.8%) |  |
| 50 + | 7 (70.0%) | 17 (12.1%) |  |
| Country of birth |  |  | 0.099 |
| Nordic countries | 8 (80.0%) | 96 (68.1%) |  |
| Europe (non-Nordic) | 2 (20.0%) | 10 (7.1%) |  |
| Other | 0 (0.0%) | 35 (24.8%) |  |
| Education |  |  | 0.64 |
| < 9 years | 1 (10.0%) | 23 (16.3%) |  |
| 9-12 years | 4 (40.0%) | 53 (37.6%) |  |
| >12 years | 5 (50.0%) | 44 (31.2%) |  |
| Missing | 0 (0.0%) | 21 (14.9%) |  |
| Neurologic diagnosis before or at baseline | 5 (50.0%) | 21 (14.9%) | 0.004 |
| Psychiatric diagnosis before or at baseline | 3 (30.0%) | 33 (23.4%) | 0.64 |

**Supplementary table 3.** Causes of death among patients with Wilson’s disease.

| **Primary cause of death** | **n** |
| --- | --- |
| Wilson's disease | 4 |
| Cardiovascular disease | 2 |
| Liver cancer | 1 |
| Neurologic disease | 1 |
| Cancer | 1 |
| Lung disease | 1 |
| **Secondary causes of death (0-5 per patient)** |  |
| Wilson's disease | 2 |
| Liver disease | 2 |
| Infection | 1 |
| Lung disease | 1 |
| Neurologic disease | 1 |
| Cardiovascular disease | 1 |
| Aspiration | 1 |
| Cancer | 1 |
| Type 2 diabetes | 1 |
